# Prevalence of abuse among the elderly population of Syangja, Nepal

**DOI:** 10.1186/s12889-021-11417-0

**Published:** 2021-07-08

**Authors:** Shiva Raj Acharya, B. K. Suman, Sandip Pahari, Yong Chul Shin, Deog Hwan Moon

**Affiliations:** 1 Graduate School of Public Health, Busan Medical Campus, Inje University, Busan, South Korea; 2 La Grandee International College, Pokhara, Nepal; 3 School of Health and Allied Sciences, Pokhara University, Pokhara, Nepal; 4 Department of Occupational Health & Safety, Inje University, Gimhae, South Korea

**Keywords:** Elder abuse, Neglect, Psychological abuse, Physical abuse, Sexual abuse

## Abstract

**Background:**

Elder abuse is recognized as a serious public health concern and top priority aging issues. World Health Organization reported that around 1 out of 6 old people in the world experienced some form of abuse. This study was carried out to find out the prevalence of different forms of abuse among elderly Nepalese people.

**Methods:**

The cross-sectional, quantitative analytical study was carried out among 373 elders of the Syangja district of Nepal. The study population was selected through simple, proportionate, and systematic sampling methods. Data was collected through face-to-face interviews using a structured questionnaire.

**Results:**

The majority of participants were female (54.5%). The prevalence of elderly abuse was found to be 54.5%. The most common form of abuse among the elderly population was neglect (23.1%), psychological abuse (20.6%), physical abuse (6.5%), financial abuse (2.4%), and sexual abuse (1.9%). Elderly females were significantly more likely to experience physical and psychological abuse.

**Conclusion:**

More than half of the elderly experienced at least one form of abuse. Neglect was found to be the most common form of abuse. The abuse was prevalent among elderly who were ill and with the habit of tobacco and alcohol consumption.

## Introduction

World Health Organization (WHO) characterizes elder abuse as a solitary or rehashed act, or absence of proper activity, happening inside any relationship where there is an assumption for trust which makes mischief or misery a more seasoned individual. The World Health Organization recognizes five sorts of elder abuse: physical, psychological or emotional, sexual, financial and intentional or unintentional forms of neglect [1, 2]. The older people feel more forlorn than other age groups due to physiological changes, an increase in psychological illnesses, social segregation due to alienation from associates and companions, reduced social movement, income, the passing of family members, and being constantly away from kids. When it comes to social improvements in human social orders, such as increased poverty, it’s reasonable to assume that the risk of elder abuse will increase [3–5]. Elder abuse may be triggered by family members, or it may be the result of a lack of planning on the part of social and health organizations to meet the needs of the elderly [6, 7].

It is projected that the elderly population of age above 60 years will grow from 900 million in 2015 to 2 billion in 2050 [4]. Despite the large population of elderly people in developed nations, population growth is believed to be seen mainly in developing countries. It is estimated that the elderly population will hit 10.5% in 2025 and 21.7% in 2050 [4]. Elder abuse and neglect global prevalence was measure at 3.2% to 27.5% [8]. The prevalence of elder abuse across the Asian region varies significantly, with estimates ranged from 0.22 per 1000 to 62% [9].

Nepal is categorized as a developing nation in the world. There is still an absence of data and realities accessible about the more established older adults of Nepal [10, 11]. According to the 2011 census of Nepal, there were 2.1 million elders, which comprised 8.1% of the nation’s absolute populace. Percent of elderly occupants during the years 1951 (5.0%), 1991 (5.8%), 2001 (6.5%), and in 2011 (8.1%) which shows that there has been a sharp expansion in the number of elder people between the year of 2001 and 2011 [12]. This shows the beginning of ageing dynamics in Nepal, which will affect Nepalese social structure and economy over the long haul [13, 14].

The annual elderly population growth rate is 3.39% which is higher than the yearly population development rate, so we can gauge that the quantity of elderly populations in Nepal is expanding [12]. The family structure in Nepal is additionally changing from reaching out to the family unit, so these elderly populations are dodged by the children and left behind alone in their home. Because of these conditions, elderly groups are confronting different kinds of social and health issues. Elder abuse can lead to a range of negative health effects in seniors, including mental illnesses, suicidal attempts, trauma, and even death [6, 14–16].

Study on elderly people is at the very initial phase and rare in Nepal. Although the government of Nepal government has been providing old-age allowances, still, care of the elderly is considered as a private family matter [17]. In developing countries, there are still a few concrete strategies for program development and facilities to increase the well-being and quality of life of the elderly [18, 19]. Elderly people being a largely neglected section of the society mostly live in a rural area of Nepal. Care of the elderly is also one of the priority areas of the Nepal Health Research Council (NHRC). The Government of Nepal has also adopted the National Policy on Aging in 2014 since its 9th and 10th long-term plan [20, 21]. This research study attempts to investigate the prevalence of the abuse among elderly population of Nepal.

## Methodology

### Study design & population

This study was a cross-sectional analytical study conducted among 373 elderly of Biruwa Rural Municipality, Syangja District, Nepal. The total elderly population above 60 years of Biruwa Rural Municipality was 2935 [22]. Elderly people of the age group above 60–80 years were included in this study. People who were unable to complete the interview process due to health conditions and personal reasons were excluded from this study.

### Sampling technique & sample size

The sample size was calculated using the formula,

$$ n=\frac{Z^2 pq N}{d^2\left(N-1\right)+{Z}^2 pq} $$

Z = standard normal variable at 95% CI (1.96), N = Total number of older adults (2935), p = estimated proportion of abuse (*p* = 0.474) based on the similar study [23], q = 1-p, d = desirable error (5%). With the application of 10% non-response rate, the sample size of this study was 373.

**Fig. 1:**
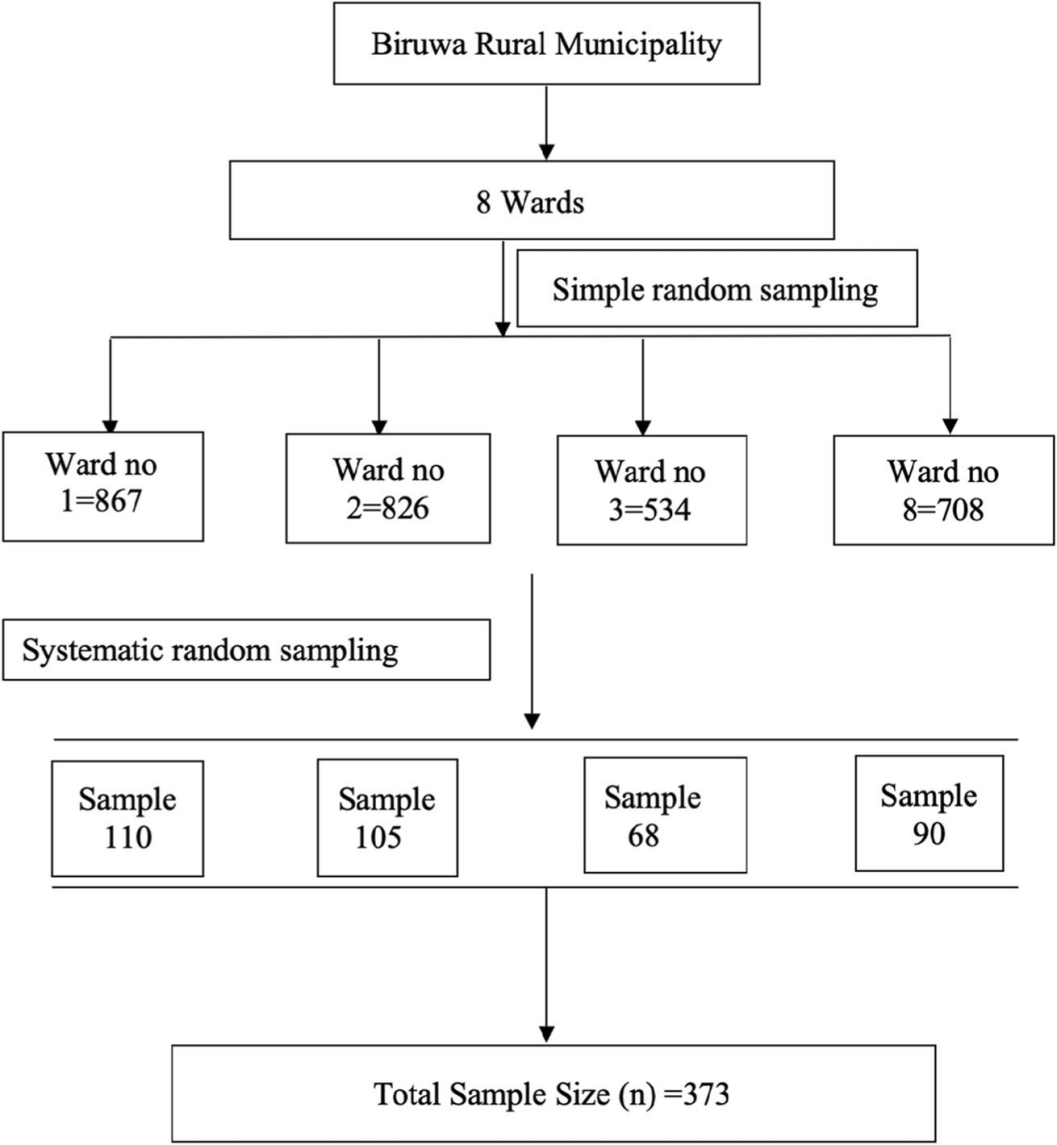
Sampling technique

Four wards of Biruwa Rural Municipality, Syangja district, Nepal were selected using simple random sampling. (Fig. 1).

### Data collection & management

The study period was between June to November, 2019. A structured interview schedule was used for data collection. The structured questionnaire included sociodemographic information (12 questions), behavior-related information (5 questions), abuse-related information (39 questions). The tool was developed based on the literature review and validated tools [23–27]. Pre-test was done among 10% of the sample. It was conducted in Arjunchaupari Rural Municipality because of neighboring rural municipality with similar characteristics. The questionnaire was pretested and the Cronbach’s alpha value for the tool was 0.71, which is within the acceptable level [28]. Data were entered in EpiData 3.1 and analyzed by using SPSS version 21. Based on the objective of the study, data was analyzed using descriptive and analytical statistics. Descriptive data analysis was carried out in terms of frequency and percentage. Bivariate analysis was performed to find out the factors associated with different forms of abuse among elderly people.

Sex, ethnicity, family type, education level, income and marital status were assessed as socioeconomic variables. Tobacco and alcohol consumption were assessed as behavior related variable. Proportion and forms of abuse were considered as dependent variables for this study.

Ethical approval was obtained from Nepal Health Research Council (NHRC) (Reg. no. 578/2019). Privacy was maintained during data collection from each participant by taking the data in a separate place, away from the family members and coding the responses. Obtained data was solely used for the study purpose. Verbal and written informed consent was taken from the respondents.

### Operational definitions

The following forms of abuse have identified [23, 25, 29];

#### Abuse

Abuse is defined as a cruel and violent treatment of a person.

#### Financial abuse

Financial abuse includes misuse of property or money. i.e. forcing an older person to sign in any document.

#### Neglect

Neglect means elder being left alone, isolated or forgotten, withholding of items necessary for daily living. i.e. not providing food, housing or medical care.

#### Physical abuse

Physical abuse is defined as the use of physical force that may result in physical pain or impairment. i.e. hitting, pushing, beating, burning etc.

#### Psychological or emotional abuse

Psychological or emotional abuse involves harassing, use of slang words, humiliating or use of gesture language. i.e. controlling behaviors, name-calling, confinement and isolation etc.

#### Sexual abuse

Sexual abuse means sexual contact without consent. i.e. unwanted touching, rape, sexually explicit photographing etc.

## Results

**Fig. 2:**
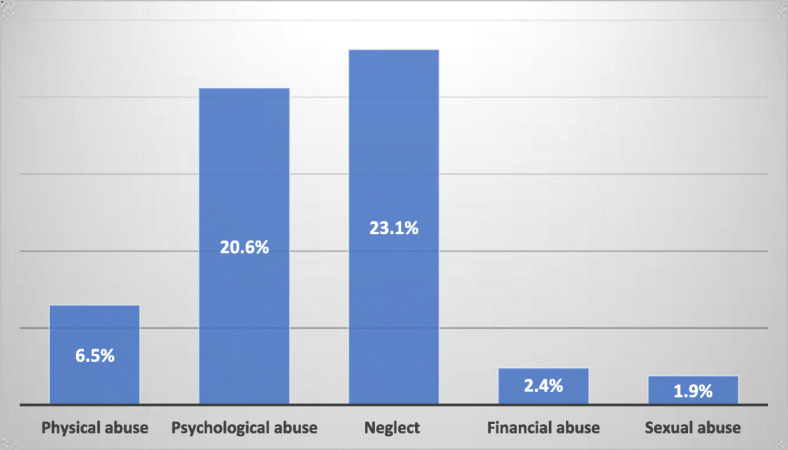
Prevalence of elderly abuse

Findings show the prevalence of elderly abuse was 54.5%. The most common elder person’s abuse was neglect (23.1%), psychological abuse (20.6%), physical abuse (6.5%) and financial abuse (2.4%) (Fig. 2). Sexual abuse was 1.9%. Nearly three-fourths (74.3%) of the participant were from the age group 60–70 years. More than two-fifth (42.6%) were Janajati. The majority of the participants were from joint family (61.1%) and engaged in agricultural works (64.3%). More than three-fourth (82.8%) of the respondents were illiterate. Of the studied population, 58% of the respondent had a habit of tobacco consumption. (Table 1).

**Table 1:** Socio-demographic characteristics of participants and abuse proportion

Variables	Frequency (%)
Age	
60–69 yrs.	277 (74.3)
70–80 yrs.	96 (25.7)
Sex	
Male	167 (44.8)
Female	206 (55.2)
Ethnicity	
Brahmin	79 (21.2)
Chhetri	77 (20.6)
Janajati	159 (42.6)
Dalit	58 (15.5)
Family Type	
Nuclear	134 (35.9)
Joint	228 (61.1)
Extended	11 (2.9)
Educational status	
Illiterate	309 (82.8)
Informal education	10 (2.7)
Basic level	37 (9.9)
Secondary level	15 (4.1)
Graduate	2 (0.5)
Marital Status	
Married	302 (81.0)
Divorced	2 (0.5)
Widow	52 (13.9)
Widower	17 (4.6)
Occupation	
Unemployed	86 (23.1)
Housewife	11 (2.9)
Agriculture	240 (64.3)
Government services	1 (0.3)
Retired	22 (5.9)
Non-government services	1 (0.3)
Business	12 (3.2)
Household Income (NRs/mon)	
Below 5000	11 (2.9)
5001–25,000	199 (53.4)
25,001–45,000	143 (38.3)
45,001–65,000	14 (3.8)
Above 65,000	6 (1.6)
Respondent’s Income (NRs/mon)	
Below 5000	322 (86.1)
5001–15,000	23 (6.3)
15,001–25,000	10 (2.7)
25,001–35,000	14 (3.8)
Above 35,000	4 (1.1)

*1USD = 111 Nepali Rupees (NRs).*

Elderly females, elderly smokers, and Dalit caste participants were all substantially more likely to be physically abused. Among the participants, tobacco users were much more likely to be sexually abused. (Table 2) Participants who had been sick in the previous year and females were subjected to experience psychological abuse. Financial abuse was significantly influenced by the participants’ education level, occupation, and income (*p* < 0.05). (Table 3).

**Table 2:** Factors associated with physical and sexual abuse

Variables	Physical abuse		*p*-value
	No	Yes	0.001*
Sex			
Male	163 (97.6%)	4 (2.4%)	
Female	186 (90.3%)	20 (9.7%)	
Ethnicity			0.001*
Brahmin	77 (97.5%)	2 (2.5%)	
Chhetri	76 (98.7%)	1 (1.9%)	
Janajati	149 (93.7%)	10 (6.3%)	
Dalit	47 (81.0%)	11 (19.0%)	
Type of family			0.240
Nuclear	127 (94.8%)	7 (5.2%)	
Joint	213 (93.4%)	15 (6.6%)	
Extended	9 (81.8%)	2 (18.2%)	
Tobacco consumption			0.027*
No	153 (96.8%)	5 (3.2%)	
Yes	196 (91.2%)	19 (8.8%)	
Alcohol consumption			0.380
No	225 (94.5%)	13 (5.5%)	
Yes	124 (91.9%)	11 (8.1%)	
Sexual Abuse			
	No	Yes	0.467
Sex			
Male	165 (98.8%)	2 (1.2%)	
Female	201 (97.6%)	5 (2.4%)	
Ethnicity			0.191
Brahmin	76 (96.2%)	3 (3.8%)	
Chhetri	76 (98.7%)	1 (1.3%)	
Janajati	158 (99.4%)	1 (0.6%)	
Dalit	56 (96.6%)	2 (3.4%)	
Tobacco consumption			0.012*
No	154 (97.5%)	4 (2.5%)	
Yes	196 (91.2%)	19 (8.8%)	
Alcohol consumption			0.231
No	226 (95.0%)	12 (5.0%)	
Yes	124 (91.9%)	11 (8.1%)	
Illness in last year			0.697
No	240 (98.4%)	4 (1.6%)	
Yes	126 (97.7%)	3 (2.3%)	

*statistically significant at 5% level of confidence

**Table 3:** Factors associated with psychological abuse, neglect and financial abuse

Variables	Psychological abuse		p-value
	No	Yes	0.001*
Sex			
Male	142 (85.0%)	25 (15%)	
Female	154 (74.8%)	52 (25.2%)	
Ethnicity			0.102
Brahmin	65 (82.3%)	14 (17.7%)	
Chhetri	65 (84.4%)	12 (15.6%)	
Janajati	128 (80.5%)	31 (19.5%)	
Dalit	38 (65.5%)	20 (34.5%)	
Tobacco consumption			0.115
No	133 (84.2%)	25 (15.8%)	
Yes	163 (75.8%)	52 (24.2%)	
Alcohol consumption			0.952
No	188 (79%)	50 (21%)	
Yes	108 (80%)	27 (20%)	
Illness in last year			0.001*
No	206 (84.4%)	38 (15.6%)	
Yes	90 (69.8%)	39 (30.2%)	
Neglect			
	No	Yes	
Age			
60–69	216 (78%)	61 (22%)	0.420
70–80	71 (74%)	25 (26%)	
Sex			0.177
Male	134 (80.2%)	33 (19.8%)	
Female	153 (74.3%)	53 (25.7%)	
Number of children			0.055
1–3	39 (65%)	21 (35%)	
> 3	251 (80.1%)	62 (19.9%)	
Financial abuse			
	No	Yes	
Sex			0.001*
Male	158 (94.6%)	9 (4.2%)	
Female	206 (100%)	0 (0%)	
Educational status			0.024*
Illiterate	305 (98.7%)	4 (1.3%)	
Informal education	10 (100%)	0 (0%)	
Basic level	34 (91.9%)	3 (8.1%)	
Secondary level	13 (86.7%)	2 (13.3%)	
Graduate	2 (100%)	0 (0%)	
Occupation			0.030*
Unemployed	84 (97.7%)	2 (2.3%)	
Housewife	11 (100%)	0 (0%)	
Agriculture	237 (98.8%)	3 (1.2%)	
Government services	1 (100%)	0 (0%)	
Retired	18 (81.8%)	4 (18.2%)	
Non-government services	1 (100%)	0 (0%)	
Business	12 (100%)	0 (0%)	
Household income (NRs/mon)			0.021*
Below 5000	10 (90.9%)	1 (9.1%)	
5001–25,000	198 (99.5%)	1 (0.5%)	
25,001–45,000	138 (96.5%)	3 (3.5%)	
45,001–65,000	12 (85.7%)	2 (14.3%)	
Above 65,000	6 (100%)	0 (0%)	
Respondents income (NRs/mon)			0.001*
Below 5000	265 (98.9%)	3 (1.1%)	
5001–15,000	22 (95.7%)	1 (4.3%)	
15,001–25,000	10 (100%)	0 (0%)	
25,001–35,000	10 (83.3%)	2 (16.7%)	
Above 35,000	3 (75%)	1 (25%)	

*statistically significant at 5% level of confidence

*USD = 111 Nepali Rupees (NRs).*

## Discussion

Our study attempts to understand more about how elderly people are abused in the community. We anticipate that this kind of research would be beneficial to policy implications to foster a healthier society. This study was conducted in Syangja district where majorities of young people immigrate abroad for the purpose of work. As a result, the majority of the elderly are living single or with their daughters-in-law and grandchildren. Furthermore, our study shows 19% of the elderly were divorced and widowed which may result in elderly abuse [23, 25, 26].. Nepal is in the midst of a demographic shift. The proportion of elderly people has been steadily increasing in the last decade due to a fall in fertility and an improvement in mortality among older people. The elderly population is also projected to grow exponentially [13]. According to our findings, 203 (54.5%) of the elderly have been reported to some forms of abuse which is slightly higher in comparison to other studies [15, 19, 23, 25, 29]. This could be due to a difference in research settings. In contrast, another study performed among the elderly residing in Pokhara, Nepal found a high prevalence of abuse (65.6%) [26].

There are various research reports focused on elderly abuse in Western countries [18, 29]. However, limited research has been done on this topic in developing countries like Nepal. In developing nations, the prevalence of abuse is considerably higher. According to a survey from Thailand and India, 50% [30] and 60% [31] of the elderly had been victims of abuse respectively. Similarly, a study from Africa shows more than 60% of elderly have reported being abused [32]. This disparity in elder abuse may be due to cultural differences, social norms, and health resource variations [10, 23, 33].

Our study found neglect (23.1%), psychological abuse (20.6%), physical abuse (6.5%), financial abuse (2.4%), and sexual abuse (1.9%) which is quite lower to studies from the eastern region of Nepal [23, 34]. The proportion of neglect is double [25, 34] as compared to our study. Since these studies were conducted among the elderly people from the urban area, where family members of the elderly were busy with their jobs and unable to find adequate time because of their office work. A study among Mexican older women showed psychological abuse (30.5%), financial abuse (8.2%), neglect (5.1%), physical abuse (3.5%) and sexual abuse (1.2%) [16]. The proportion of neglect is less by one third as compared to our study. This may be attributed to the fact that the sample only had female participants. Furthermore, to ensure the long-term wellbeing of the elderly, the government should undertake a nationwide initiative to establish an “Elder Service Center” in each municipality [23, 25, 26, 35].

Nepal is a largely male-dominated society where women’s rights are underappreciated. Even in married couples in Nepal, male partners have a dominant position in forming sexual relations, regardless of their partner’s desires which may eventually the underlying factor for the sexual abuse in the community [25, 32]. Elderly abuse was found to be prevalent among participants who were female, illiterate, belonged to the Dalit caste group, low financial situation, tobacco users, and poor health. These results agree with those of several other studies [25, 31, 32, 36, 37]. Other research have shown that elderly people who were ill are more likely to be abused [19, 38]. Elders from the Dalit population were more likely to experience abuse than those from higher castes, which may be due to the poor socio-cultural and socio-economic status of the caregiver. Community awareness and educational interventions such as self-motivation, orientation about elder abuse should be given for the prevention of elder abuse before reaching an advanced age [25, 31]. Our study consistently suggest that isolation and a lack of social support were important risk factors for elder abuse which is supported by the study conducted in Malaysia [39]. Based on our study findings, love and care to the elderly people from the family members and access to quality health services to older people should be provided. Furthermore, the health status of elderly people should be monitored timely [15, 19, 26, 32]. Study shows more than one third (34.6%) elderly were suffered from the illness in the last year.

Despite the government of Nepal’s many measures in support of older persons, compliance with current national plans and policies, global and regional resolutions and pledges remains a challenge. Protection and possibilities for the elderly aren’t well-initiated, but are instead embraced through conventional ideas such as mercy and enforced care. The government’s programs were principally set up to protect elder citizens’ rights to dignity and security [17, 21]. Awareness, capacity building, economic improvement, participation, health and nutrition, basic facilities and services should be increasingly focused on the optimal development of elderly communities. It is recommended that the government should take the required measures to improve the elderly’s quality of life and strengthen all aspects of their lives at the community level.

This study was conducted in a limited sample population. As this study is carried out in a small geographical area, findings cannot be generalized to the whole population of the nation. This study excludes the elderly who were ill and above 80 years. When disclosing elderly abuse, participants may have certain biases. A face-to-face interview was used to collect data, which may lead to question biases.

Our study findings were shared with the local government so that appropriate steps could be taken to address the issue of elder abuse in the sampled community. Furthermore, discussion regarding the mitigation of elder abuse and planning of appropriate community-based awareness programs were also performed to address this issue.

## Conclusion

Elderly abuse is still highly prevalent in a rural community of Nepal. Neglect was found to be the most common form of abuse followed by psychological abuse, physical abuse, financial abuse, and sexual abuse. Addressing the specific ethnic issues (Dalit caste community) and lower socioeconomic status has a significant implication to improve the well-being of the elder people. Implementation of community-focused development programs and policies is highly recommended. A further in-depth investigation is needed to discover more about the real nature of elderly abuse in Nepal.

## Data Availability

The datasets generated and/or analyzed during the current study are not publicly available due to the privacy and confidentiality of the participant. As we mentioned that the data will be only use for study purpose, not for the public availability during the informed consent. The datasets analyzed during the current study are available from the corresponding author on reasonable request.
